# Acquired Elliptocytosis as a Manifestation of Myelodysplastic Syndrome with Ring Sideroblasts and Multilineage Dysplasia

**DOI:** 10.1155/2017/3625946

**Published:** 2017-10-11

**Authors:** Jacob D. Kjelland, Denis M. Dwyre, Brian A. Jonas

**Affiliations:** ^1^Department of Internal Medicine, University of California Davis Medical Center, Sacramento, CA 98517, USA; ^2^Department of Pathology, University of California Davis Medical Center, Sacramento, CA 98517, USA; ^3^Department of Internal Medicine, Division of Hematology and Oncology, University of California Davis Medical Center, Sacramento, CA 98517, USA; ^4^VA Northern California Health Care System, Mather, CA 95655, USA

## Abstract

Acquired elliptocytosis is a known but rarely described abnormality in the myelodysplastic syndromes (MDS). Here we report the case of an elderly male who was admitted to the hospital with chest pain, dyspnea, and fatigue and was found to be anemic with an elliptocytosis that had only recently been noted on peripheral smears of his blood. After bone marrow biopsy he was diagnosed with MDS with ring sideroblasts and multilineage dysplasia and acquired elliptocytosis. Here we report a rare case of acquired elliptocytosis cooccurring with MDS with ring sideroblasts and multilineage dysplasia.

## 1. Introduction

The myelodysplastic syndromes (MDS) are a heterogeneous group of clonal myeloid hemopathies that result in ineffective development of one or more hematopoietic lineages. The genetic alterations and other pathologic processes in the bone marrow causing dysfunctional hematopoiesis lead to a wide range of disease phenotypes which can range from mild single lineage cytopenia to severe pancytopenia or transformation to acute leukemia. Similarly diverse are the microscopic characteristics of the dysplastic cells in the bone marrow and peripheral blood.

In this report, we present the case of an elderly male who was diagnosed with MDS after being admitted to our hospital for symptomatic anemia. Review of his bone marrow biopsy showed two morphological findings of particular note. The first finding was ring sideroblasts, which are present in about 25% of cases of MDS [1]. Ring sideroblasts are erythroid precursor cells in which iron is unable to be exported from the mitochondria due to one of a variety of defects. This leads to an excess of iron within the mitochondria, which stains with Prussian blue, revealing the so-called ring sideroblast. As with most findings in MDS, ring sideroblasts are not specific to MDS but are found in numerous congenital and acquired conditions encompassing many genetic and acquired defects. Recently, mutations in a gene coding for a portion of the U2 snRNP have been linked to the development of ring sideroblasts in MDS. In one study,* SF3B1*, located on chromosome 2 (http://genome.ucsc.edu) was found to be mutated in 20% of all MDS cases and 65% of those with ring sideroblasts [2]. In another recent study, 81% of MDS subtypes with ring sideroblasts had* SF3B1* mutations [3]. It has been speculated that the mutated protein may cause generalized downregulation of several core pathways within the mitochondria, potentially accounting for the higher frequency of ring sideroblasts observed in patients with mutated* SF3B1* [2]. The second finding in our case was elliptocytosis, a finding which can be hereditary or can be acquired. Hereditary elliptocytosis is caused by mutations in in structural components of the erythrocyte cell membrane. Mutations in genes coding for protein 4.1, alpha spectrin, beta spectrin, band 3, and Glycophorin C have all been implicated in hereditary elliptocytosis [4, 5]. Of note, other hereditary conditions, such as sickle cell anemia, thalassemias, and pyruvate kinase deficiency can have elliptocytosis as a feature [4]. Acquired elliptocytosis is sometimes seen in myeloid malignancies (including MDS) as well as in iron deficiency anemia, megaloblastic anemias, polycythemia, and myelofibrosis [5].

## 2. Case

A 70-year-old Caucasian male immigrant from Eastern Europe with a 6-year history of anemia and an 8-year history of thrombocytopenia was admitted to our hospital for chest pain and dyspnea. At the time of his admission, his exam was notable for mild scleral icterus and a palpable spleen 2 cm below the costal margin.

He had undergone prior workup for his cytopenias without a clear diagnosis. There was no family history of anemia. He did not use alcohol. His medications included amiodarone and ranolazine (medications associated with bone marrow suppression), but no other medications known to affect hematopoiesis. Records obtained documenting the outpatient workup included studies showing normal vitamin B12 and folate levels, decreased haptoglobin with a negative Coombs test, and a bone marrow biopsy performed 2 years earlier which showed a hypercellular marrow, moderate erythroid hyperplasia, a deletion of chromosome 20q by cytogenetics, 3-4% ring sideroblasts, and normal flow cytometry studies. Based on these findings, there was concern for a pre-MDS state, such as clonal cytopenia of undetermined significance (CCUS). One month prior to admission, a peripheral smear showed elliptocytosis. Physician's notes from the outpatient workup indicate that a peripheral smear was reviewed 2 years earlier and there was no mention of elliptocytosis or spherocytosis at that time.

**Figure 1:**
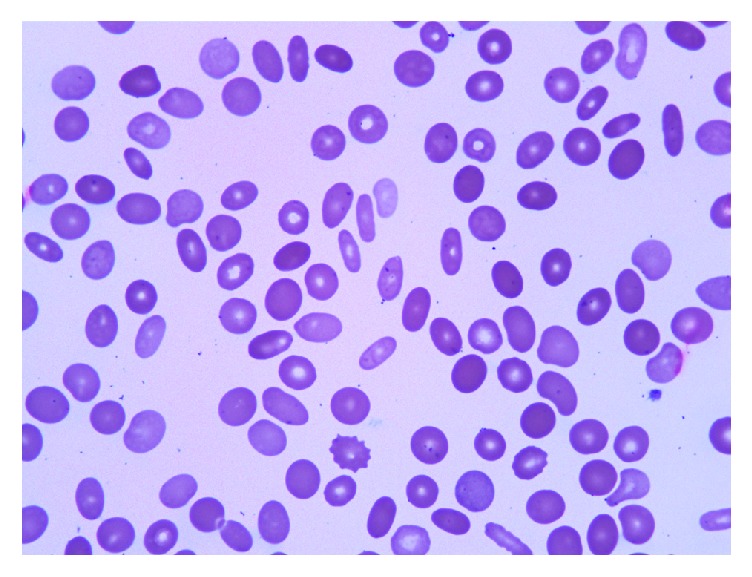
Peripheral blood smear (Wright-Giemsa stained, 100x): peripheral smear showing anemia, with numerous elliptocytes and thrombocytopenia.

**Figure 2:**
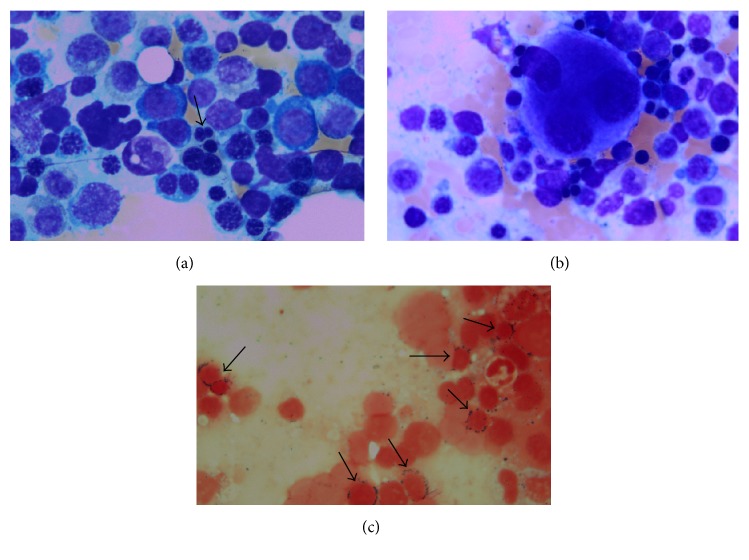
(a) Bone marrow aspirate (Wright-Giemsa stain, 100x). High magnification of the bone marrow aspirate shows a marrow with erythroid hyperplasia, megaloblastic maturation, and a dysplastic erythroid cell (arrow). (b) Bone marrow aspirate (Wright-Giemsa stained, 100x). High magnification of the bone marrow aspirate shows a dysplastic megakaryocyte with separate lobes. (c) Bone marrow aspirate (Iron stain, 100x). The iron stained aspirate shows multiple ring sideroblasts (>15%) (arrows).

At time of admission, the patient's hemoglobin was 7.6 g/dL with an MCV of 91.1 *μ*m^3^, a platelet count of 28,000/mm^3^, white blood cell count of 5,200/mm^3^, and absolute neutrophil count of 4,600/mm^3^. He had 6.7% reticulocytes with an absolute reticulocyte count of 190,000/mm^3^. His total bilirubin was elevated at 4.5 mg/dL with direct bilirubin of 2.4 mg/dL. Transaminases were normal. LDH was elevated at 349 U/L, but haptoglobin levels were normal and a direct Coombs test was negative. Methylmalonic acid level was slightly elevated at 0.48 *μ*mol/L (normal up to 0.40 *μ*mol/L). Thyroid studies and vitamin B12 levels were normal. Folate was not checked. Ferritin was 540 ng/mL, with normal serum iron and transferrin levels. No paroxysmal nocturnal hemoglobinuria clone was detected by peripheral blood flow cytometry. Osmotic fragility was tested and found to be normal. HIV screen was negative. Hematology was consulted for diagnostic assistance. Review of the peripheral smear showed marked elliptocytosis (Figure 1). A bone marrow biopsy was performed which showed a hypercellular marrow with erythroid hyperplasia, dyserythropoiesis and dysmegakaryopoiesis, and adequate stainable iron with >15% ring sideroblasts and 3% blasts (Figure 2). Cytogenetic analysis showed 46,XY,del(20)(q11.2q13.3)/47,idem,+del(20) [6].

Based on these findings, the patient was diagnosed with MDS with ring sideroblasts and multilineage dysplasia (MDS-RS-MLD). His IPSS-R score was 5.5, stratifying him to the high risk IPSS-R group, with an estimated median overall survival of 1.6 years [7]. During his hospital stay he received a total of 2 units of packed red blood cells and 1 unit of platelets, with resolution of his chest pain and dyspnea. Prior to this time, he had not required transfusion for his marrow disorder and had only received blood product transfusions in the postoperative setting (distant open heart surgery and others). He was discharged on hospital day 6 with a follow-up appointment in hematology clinic scheduled. He rescheduled the appointment for unknown reasons and, unfortunately, passed away at home approximately three weeks after discharge. No autopsy was performed.

## 3. Discussion

Over the last 4 decades, understanding of the pathophysiology of MDS has advanced considerably. As a result, there have been revisions in classification and risk scoring of MDS leading to improvements in the ability of clinicians to offer a more accurate prognosis for affected patients. Still, with the considerable heterogeneity and incomplete understanding of MDS, additional investigations into the mechanisms underlying the development of MDS will likely yield new information and insight into disease progression and refinements to MDS classification.

This case report describes a unique case of acquired elliptocytosis presumably related to MDS, with a high percentage of ring sideroblasts. MDS with ring sideroblasts is a relatively common subtype of MDS, found in approximately 25% of all MDS cases [1]. Acquired elliptocytosis in MDS, however, is rare. Only 14 such cases are available in the literature to date (Table 1). According to the 2016 WHO guidelines, a majority of previously reported cases of acquired elliptocytosis in MDS would be classified as either MDS with single lineage dysplasia or multilineage dysplasia (10 of 14 cases) [8]. The case reported in this paper is classified as MDS with ring sideroblasts and multilineage dysplasia (MDS-RS-MLD) and is now the 15th documented case of acquired elliptocytosis in MDS. Of the 15 cases of MDS with an acquired elliptocytosis, 14 reported cytogenetics, and, of those 14 patients, 11 had deletions of chromosome 20q.

While the causal factor(s) leading to acquisition of elliptocytosis in cases of MDS have not been definitively identified, several cases have noted decreased levels of human erythroid protein 4.1 in association with del(20q) [9–11]. This protein is a major cytoskeleton component in erythrocytes and reductions in its levels are implicated in a small percentage of cases of hereditary elliptocytosis [4, 5]. Interestingly, the* EPB41* gene, which codes for human erythroid protein 4.1 is found on chromosome 1 (http://genome.ucsc.edu) [9]. Since the common location of lost genetic material in most of the cases of MDS with acquired elliptocytosis is on chromosome 20, it has been hypothesized that the association between del(20q) and reduced levels of human erythroid protein 4.1 is a consequence of loss of factors affecting transcription or splicing of the* EPB41* gene's mRNA [9]. Of note, among the 14 known patients with MDS and acquired elliptocytosis who have cytogenetic data available, only one had a chromosome 1 abnormality and this patient also had a deletion on chromosome 20 [12]. We can speculate that there may be a role for cooperating mutations, possibly involving splice factors such as the one coded for by* SF3B1* and downregulation of* EPB41* or another gene coding for an erythrocyte membrane protein, leading to peripheral elliptocytosis in MDS. Such a mechanism would provide an explanation for the development of elliptocytosis in MDS and at the same time link the seemingly unrelated observations of decreased levels of human erythroid protein 4.1 and deletion of chromosome 20q in MDS with acquired elliptocytosis [9–11]. To evaluate this idea, future cases of MDS with acquired elliptocytosis should be studied using techniques such as next-generation sequencing based myeloid mutation profiling, RNA-sequencing to evaluate mRNA expression, and gene expression profiling.

A correlation between deletions on the long arm of chromosome 20 and acquisition of elliptocytosis in MDS has emerged since the first patient with acquired elliptocytosis in what we now call MDS was reported by Hartz et al. in 1984 (then called preleukemic syndrome) and the first such case where cytogenetic studies were performed by Rummens et al. in 1986 [13, 14]. The focus of this report is on a rare and interesting finding, but it should be noted that chromosome 20 deletions are not uncommon in MDS. Chromosome 20 deletions were found in 7% of MDS cases in one large case series and are common enough to be included as a variable in the IPSS-R for MDS [7, 15]. While del(20q) has been shown to confer good cytogenetic risk, the impact of acquired elliptocytosis on prognosis is unknown and may benefit from further study [16]. The observation that acquired elliptocytosis seems to be related to del(20q) in MDS leads one to question why acquired elliptocytosis is so rare, given that 20q deletions are relatively common in MDS. To our knowledge, this question has yet to be addressed. Future research aimed at resolving this paradox may help to further refine the categorization or risk stratification for patients with MDS if a causative link between del(20q) and acquired elliptocytosis can be established.

## Figures and Tables

**Table 1:** Characteristics of the current and previously published cases of myelodysplastic syndrome with acquired elliptocytosis.^*∗*^

Age and gender	FAB classification	2016 WHO classification	Cytogenetics	IPSS-R score	Reported outcome	Reference number
81, male	RA	MDS-like CMML	46,XY,i(14)(q10)[5]/46,XY[35]	3-4 (blasts <5% but may be >2%)	NR	[6]
67, female^†^	RA-EB	MDS-EB-2	del(20)(q11q13)[20]	5	NR	[17]
72, male	RA	MDS-MLD	NR	0–5 (cytogenetics NR, blasts <5% but may be >2%)	Death, 8 years after diagnosis	[13]
75, male	RA	MDS-MLD	Del(20q)(q11.2)	2-3 (blasts <5% but may be >2%)	NR	[18]
59, male	RA	MDS-MLD	44, XY; -3, -5, 12p+, -15, 17p and 45, XY; -5, 12p+, -15,17p	7	NR	[14]
60, male	RA-EB	MDS-EB-1	Del(20q)	4	Progressed to AML within 1 year of MDS diagnosis, death 1 year later	[14]
82, male	RA	MDS-SLD or MLD	46 XY, del(20q)(q11.2)	1.5–3 (blasts <5% but may be >2%, ANC NR)	NR	[19]
79, male	RA	MDS-SLD or MLD	46 XY, del(20q)(q11.2)	1.5–2.5 (blasts <5% but may be >2%)	NR	[19]
78, male	RA	MDS-SLD or MLD	Del(20)(q11.2)	1-2 (blasts <5% but may be >2%)	NR	[9]
74, female	RA	MDS-SLD or MLD	t(14)	3-4 (blasts <5% but may be >2%)	NR	[9]
77, male	RA	MDS-SLD or MLD	Del(20q)	1-2 (blasts <5% but may be >2%)	NR	[9]
66, male	RA	MDS-U or CCUS	46,XY,del(20)(q11.2)[20]	2.5–3 (ANC NR)	Progressed to RA-EB, achieved remission with therapy	[10]
59, male	RA	MDS-MLD	Del(20q)	2.5–4.5 (platelets NR, blasts <5% but may be >2%)	NR	[11]
72, male	RA	MDS-SLD	+1,der(1;5)(q10;p10),t(1;5)(p10;q10),del(20)(q11)	5	Reported to have remained well for 18 months at publication	[12]
*70, male*	*RARS*	*MDS –RS-MLD*	*46,XY,del(20)(q11.2q13.3)/47,idem,+del(20)*	*5.5*	*Death, 1 month after diagnosis*	*Currently reported case*

FAB and 2016 WHO classifications were determined using data from published cases. Where data are not sufficient to determine classification, the possible classifications based on available information are shown. IPSS-R score calculated using data from published cases. Where insufficient data is available, the range of possible scores is presented with missing information noted in parentheses. *General abbreviations*: ANC: absolute neutrophil count, MPN: myeloproliferative neoplasm, NA: not applicable, NR: not reported, *FAB abbreviations*: RA: refractory anemia, RA-EB: refractory anemia with excess blasts, RARS: refractory anemia with ring sideroblasts. *WHO 2016 abbreviations*: MDS-SLD: MDS with single lineage dysplasia, MDS-MLD: MDS with multilineage dysplasia, MDS-MLD-RS: MDS with multilineage dysplasia and ring sideroblasts, MDS-EB-1/2: MDS with excess blasts 1 or 2, MDS-U: MDS unclassifiable, AML: acute myeloid leukemia, CMML: chronic myelomonocytic leukemia, CCUS: clonal cytopenia of unclear significance. ^*∗*^One case not included in this report was classified as MDS/MPN when published in 2008 but would now be classified as AML based on 25% blasts on bone marrow biopsy. ^†^Molecular data available for this case; reported findings included no mutation in *JAK2*, *CALR*, or *MPL.*
